# ABCMdb reloaded: updates on mutations in ATP binding cassette proteins

**DOI:** 10.1093/database/bax023

**Published:** 2017-03-18

**Authors:** Hedvig Tordai, Kristóf Jakab, Gergely Gyimesi, Kinga András, Anna Brózik, Balázs Sarkadi, Tamás Hegedűs

**Affiliations:** 1 MTA-SE Molecular Biophysics Research Group, Hungarian Academy of Sciences and Department of Biophysics and Radiation Biology, Semmelweis University, Budapest 1094, Hungary; 2 Institute of Biochemistry and Molecular Medicine, University of Bern, Bern 3012, Switzerland and; 3 Institute of Enzymology, Research Centre for Natural Sciences, Hungarian Academy of Sciences, Budapest 1117, Hungary

## Abstract

ABC (ATP-Binding Cassette) proteins with altered function are responsible for numerous human diseases. To aid the selection of positions and amino acids for ABC structure/function studies we have generated a database, ABCMdb (Gyimesi *et al.*, ABCMdb: a database for the comparative analysis of protein mutations in ABC transporters, and a potential framework for a general application. *Hum Mutat* 2012; 33:1547–1556.), with interactive tools. The database has been populated with mentions of mutations extracted from full text papers, alignments and structural models. In the new version of the database we aimed to collect the effect of mutations from databases including ClinVar. Because of the low number of available data, even in the case of the widely studied disease-causing ABC proteins, we also included the possible effects of mutations based on SNAP2 and PROVEAN predictions. To aid the interpretation of variations in non-coding regions, the database was supplemented with related DNA level information. Our results emphasize the importance of *in silico* predictions because of the sparse information available on variants and suggest that mutations at analogous positions in homologous ABC proteins have a strong predictive power for the effects of mutations. Our improved ABCMdb advances the design of both experimental studies and meta-analyses in order to understand drug interactions of ABC proteins and the effects of mutations on functional expression.

**Database URL:** http://abcm2.hegelab.org

## Introduction

ABC (ATP Binding Cassette) membrane proteins are molecular machines converting the binding and hydrolysis of ATP to conformational changes in the protein to relocate substrates through the membrane bilayer or regulate channel function (1–3). The function of ABC proteins is important in various physiological processes. Some members of this protein superfamily (e.g. MDR1/ABCB1, MRP1/ABCC1, BCRP/ABCG2) transport substrates with hydrophobic character, such as lipids, hormones and xenobiotics (4). TAP1 and TAP2 transporters associated with antigen processing catalyze the movement of small peptides from the cytosol to the endoplasmic reticulum for presentation by the MHC-I complex (5). ABCA1 is involved in cholesterol transport and ABCA4 transports vitamin A derivatives in rod photoreceptor cells thus participating in HDL biogenesis and vision cycle, respectively (6, 7). Interestingly, three members of the ABCC subfamily are not active transporters. CFTR (cystic fibrosis transmembrane regulator, ABCC7) exhibits cAMP-dependent chloride channel function, while ABCC8 (Sulfonylurea receptor 1) and ABCC9 (Sulfonylurea receptor 2) regulate inward-rectifier potassium ion channels and thus membrane potential changes associated with various downstream events, such as insulin secretion in pancreatic cells (8, 9).

The function and expression of these ABC proteins can be altered either by mutations or regulatory processes and their malfunction or changed expression level can lead to numerous pathological states (1, 10). Mutations in many ABC proteins have been linked to severe inherited diseases. Deleterious variations in ABCA1 and ABCA4 lead to Tangier and Stargardt diseases, respectively (6, 7). Mutations in TAPs result in immune deficiency manifested e.g. as increased risk for cancer (11). Genetic alterations in the *ABCB11* gene can cause a severe form of liver disease, progressive familial intrahepatic cholestasis type 2 (PFIC2) (12). Mutations of ABCC6 and CFTR are associated with their decreased functional expression that lead to a rare disease, pseudoxanthoma elasticum and the most frequent severe, monogenic inherited disease, cystic fibrosis (CF), respectively (8, 13). Several transporters from the ABCB, ABCC and ABCG subfamilies are multidrug transporters promiscuously recognizing different compounds with highly diverse chemical properties (3, 4). These transporters strongly influence the ADME-Tox (Adsorption, Distribution, Metabolism, Excretion and Toxicity) properties of xenobiotics including drugs. Prescribing a drug at the generally recommended dose for a patient carrying a mutation in a multidrug transporter may result in serious or even lethal increase in the side effects caused by the changed half-life and concentration of the drug in the body. For example, the Q141K variation in ABCG2 causes a decreased expression and function of this transporter thus results in increased brain accumulation of chemotherapeutic agents, such as tyrosine kinase inhibitors (14–16).

There is a great importance to understand the effects of variations in detail in order to predict the severity of a disease, or to apply a drug at a different concentration than the usual recommended dose. For these reasons, gene variations are collected and annotated from many sources in various databases. dbSNP has been created to provide a central repository for single base nucleotide substitutions, short deletions and insertions (17). There are also annotations on the type of mutations (e.g. stop gained, frame shift) of polymorphisms. To specifically report the relationships among human variations and phenotypes with supporting evidence, the ClinVar database has been established and tightly coupled to dbSNP (18). In parallel, coexisting with these large genomic databases, many locus specific databases (LSDBs) are maintained by experts of the given field and these are usually manually annotated. Therefore some of these LSDBs contain rich data on variations, sometimes including detailed clinical data, thus are the primary information sources. LSDB installations are listed at several websites including the Human Variome Project site (http://www.hgvs.org/locus-specific-mutation-databases) and the LOVD (Leiden Open Variation Database) site (http://grenada.lumc.nl/LSDB_list/lsdbs) (19).

For diagnosis and personalized treatment, the level of deleteriousness of a variation might be sufficient. However, for rational drug development, to understand the mechanism of alteration is necessary, as stability, localization, or function of the protein may be variably altered by a variation. Information on these phenomena may not be available or difficult to gather from experimental studies. *In silico* tools can be exploited for example to predict changes in stability (ΔΔG values) or cellular localization of the target protein. Both variation databases and tools for effect predictions are summarized in the recent review of Niroula and Vihinen (20, 21).

There are relatively few data sources with exhaustive number of variations with sufficient details on ABC proteins to support clinical or basic research projects. These include the well-known CFTR, CFTR2 and LOVD ABCC6 LSDBs. To facilitate structure/function studies of the ABC protein superfamily, we have set up a protein-centered database that contains mutations retrieved from the literature using semi-automatic methods (22). Missense and nonsense mutations altering protein sequence could have been investigated in detail in the context of sequence alignments and structural environment. Although the mutations have been extracted from full text papers and the environment of the mutation mentions were listed in the database, facilitating to explore the effects of the alterations, it is still a challenging task to assess the consequences of a variation if studying the effect was not an aim in the original publication. Therefore we supplemented our database with predictions for the effect of the variation, as well as with genomic information including gene sequences, promoters, exon/intron boundaries and experimentally determined transcription factor binding sites. We have confidence that our web application and database will become a central toolset for ABC protein research.

## Materials and methods

### Databases and sequences

Records, which contained the ‘single nucleotide variant’ annotation in the field of ‘variant_type’, were collected from ClinVar as of January 2016. Variants were set to deleterious, neutral and unspecified, when the significance/description field contained ‘pathogenic’ or ‘likely pathogenic’, ‘benign’ or ‘likely benign’ and other (‘uncertain significance’, ‘conflicting interpretations of pathogenicity’ and ‘not provided’) annotations, respectively.

ABCB11 variations were collected from two reviews (23, 24). A variant was set to deleterious when it was annotated as PFIC (progressive familial intrahepatic cholestasis) or BRIC (Benign recurrent intrahepatic cholestasis) or ICP (Intrahepatic cholestasis of pregnancy), to benign when ‘no association’ expression or no annotation and unspecified in all other cases.

Tab delimited file of ABCC6 variants were downloaded from LOVD/ABCC6 (http://www.ncbi.nlm.nih.gov/lovd/home.php?select_db=ABCC6) as February of 2016. Since the LOVD sites at NIH were closed on 30th of September, 2016, we link the downloaded MS Excel file containing the analyzed. All listed ABCC6 variations in LOVD were disease associated.

Data on mutations of CFTR were collected from the CFTR1 (http://www.genet.sickkids.on.ca/) and CFTR2 (http://cftr2.org/) databases. A mutation was set to deleterious when CF or CF with PI (Pancreatic Insufficiency) annotation was found in CFTR2, or when we could identify the disease association based on clinical notes in CFTR1. A variant was marked in our database as neutral, when no CF associated annotation was found in CFTR2. It was set to unspecified when the ‘variant consequence’ field in CFTR2 contained the ‘unknown significance’ expression and disease association could not be concluded based on CFTR1 database annotations.

**Annotations of gene level features, including** boundaries of genes, mRNAs and exons, **were collected based on the human genome assembly** GRCh38.p2 (**Genome** Reference Consortium Human Build 38 patch **release** 2). Reference mRNA sequences were gathered from the NCBI gene/nucleotide database. Since protein sequences in our database are from UniProt, mRNA after translation and protein sequences were compared. All ABC sequences matched, except two ABCA members. In the case of the ABCA2 protein sequence, EA amino acids at the positions 53–54 were replaced by EVS, which is annotated as a natural variant in UniProt. In the case of ABCA13 we use the variant K4446V corresponding to the reference mRNA sequence, which is annotated as a sequence conflict in UniProt.

### Software, scripts and analysis

Most of the data collection and analysis were performed using in house scripts written in Python or R. Python libraries BioPython (25), numpy (http://www.numpy.org) and matplotlib (http://matplotlib.org) were extensively used. Conservativeness of an amino acid replacement was determined based on the BLOSUM62 substitution matrix. Sequence alignments were generated by ClustalW and manually adjusted when needed (e.g. at the N-terminus of the nucleotide binding domains in the ABCC subfamily). Effects of mutations were predicted by SNAP2 (26) and PROVEAN (27).

## Results and discussion

### Integration of data on the effects of variations

Although it would be essential to know the detailed effect of a missense mutation, whether it alters the function, folding, or targeting of a protein, current predictors, data mining applications and databases cannot deliver such fine grade answers. Therefore we aimed to collect and integrate annotations on the deleterious and neutral effects of variations from the widely used and curated ClinVar database. We especially looked for variations resulting in missense or nonsense mutations. Although ABC proteins play roles in rare diseases (10, 28), there is a surprisingly low number of ABC mutations deposited in this central database (see online supplementary material for Table S1). Moreover, a large fraction of the data is unspecific about the effect of the mutations.

In order to compare different resources extensively, we have analyzed in detail and set as examples three well known disease causing ABC proteins ABCB11 (Bile Salt Export Pump, BSEP) (12, 23, 24), ABCC6 (MRP6) (13, 29) and CFTR (ABCC7) (8) mutated in progressive familial intrahepatic cholestasis, pseudoxanthoma elasticum and cystic fibrosis, respectively (10). ClinVar contained only 14 SNPs for the coding region of *ABCB11* and 21 for *ABCC6* (see online supplementary material for Table S1, Figure S1) in January 2016. In contrast, for *CFTR* ClinVar contained not only a high number of reported mutations (731), but covers most of the clinically relevant alterations of this gene (see below).

**Figure 1:**
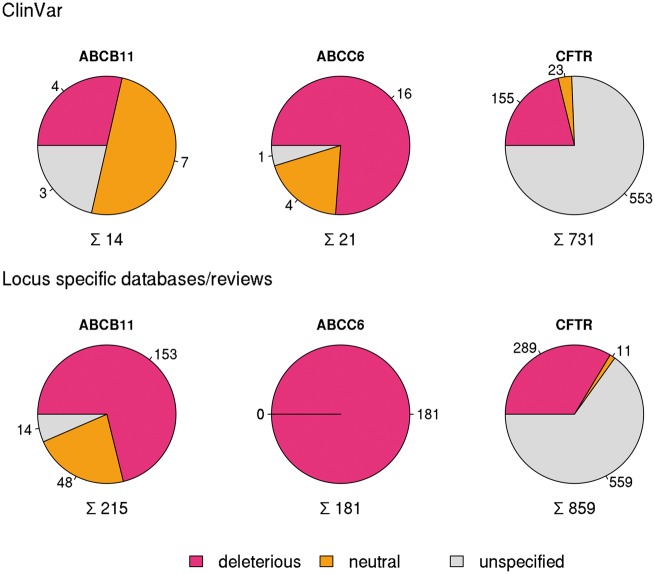
Variations in coding regions of ABCB11, ABCC6 and CFTR from ClinVar (top row), locus specific databases and reviews (bottom row).

Other large resources were found insufficient for our studies. The ExAC browser (http://exac.broadinstitute.org) provides data of sequenced individuals, but the disease status and the effects of the variations is not annotated. Results of the 1000 Genomes sequencing project (http://www.1000genomes.org) also cannot be employed, because ABC proteins play a role mainly in rare diseases, thus disease causing mutations are not expected to be detectable in the set of 3775 processed genomes (as of June 2016).

Since the above data sources contain limited amount of information on ABC protein variations, we also investigated locus specific databases (LSDBs). To identify LSDBs specific for ABC proteins we turned to the Leiden Open Variation Database server that also hosts a list of registered gene specific databases (http://www.lovd.nl/LSDBs) (19). Unfortunately, databases and data on ABC genes seem to be underrepresented. For most ABC genes only a few registered databases exist with the following types: (1) LOVD installations with curator vacancy (these are empty databases), (2) gene-specific links to large public databases including ClinVar and PharmKGB and (3) country initiatives for collecting region specific variants (e.g. Brazilian Initiative on Precision Medicine). Moreover, these installations also contain a low number of variants and low amount of information e.g. on the effect and clinical relevance of ABC gene mutations. For example, LOVD installations at the University of Melbourne (http://proteomics.bio21.unimelb.edu.au/lovd/genes/ABCB11) and Cincinnati Children's Hospital Medical Center (https://research.cchmc.org/LOVD2/home.php?select_db=ABCB11) list 13 and 114 unique variants of the *ABCB11* gene, respectively. In spite of the considerably large number of records in the latter database, there is no detailed evidence for the effects provided.

**Figure 2:**
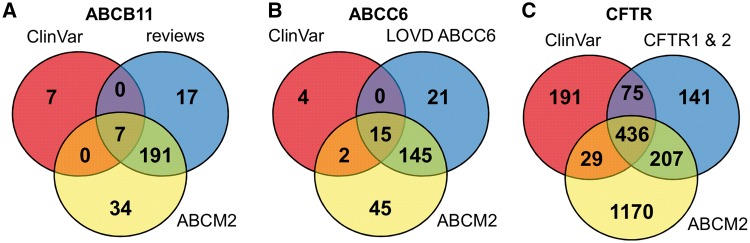
Distribution of ABCB11, ABCC6 and CFTR variants between ClinVar, ABCM2, and other resources.

To increase the number of *ABCB11* variants in our study, we also analyzed two major reviews on *ABCB11* variations and recorded 215 missense and nonsense mutations of *ABCB11* (23, 24). With relatively small efforts, 153 and 48 out of these 215 records could be identified as deleterious and neutral, respectively (Figure 1). The effects of the remaining 14 variations have not been determined. There are extensively curated databases for *ABCC6* (http://abcm2.hegelab.org/ABCC6_LOVD_20160217.xlsx) and *CFTR* (http://cftr2.org) (30). While *CFTR* is the first disease associated gene, which has been studied in great detail supported by a community of researchers and patients’ relatives, *ABCC6* has been linked more recently to pseudoxanthoma elasticum that affects a smaller population as compared to cystic fibrosis. However, the collection and long term maintenance of *ABCC6* variation data are also supported by an enthusiastic patient Group (31). The overlap between these resources and data in ClinVar varies greatly (Figure 2). One of the reasons may be technical, associated with challenges of merging a full database into ClinVar as in the case of LOVD *ABCC6*, which is being migrated to ClinVar. Figure 2 also indicates that we were able to identify a high number of mutations, which are not present in ClinVar or these locus-specific databases, since they most likely have been generated for experimental studies.

In summary, in spite of the available platforms to integrate data on variants, there is a general lack of annotated data on the variations of ABC proteins in public databases. Therefore we extended our database to contain not only semiautomatically identified mutations for ABC genes, but also curated datasets from other databases including indications for the effect of the listed variations. At this moment our ABCM2 database includes data only from ClinVar for all ABC proteins and from the above mentioned LSDBs and reviews for the three selected proteins. According to our opinion, the ABCM2 database with its limitations still may serve as a central data source for researchers in the ABC field.

### Predicting the effects of variations using *in silico* methods

As seen above, many variations have unspecified effects regarding not only the protein structure and function, but also clinical consequences. This lack of information causes serious difficulties when designing experiments or providing clinical diagnosis, while this problem may be circumvented by using *in silico* methods to predict the effects of variations. There have been a few trials to employ computational methods to assess the effects of SNPs of ABC proteins. Variations in the ABCC1 protein (MRP1) have been evaluated by SIFT and PolyPhen in the laboratory of S.P. Cole (32). Dorfman *et al.* tested PANTHER (33), SIFT (34) and PolyPhen (35) whether these tools can aid CF-associated clinical diagnosis (36). The conclusion of all of these studies was that current *in silico* methods are not sufficient to provide guidance either in planning mutagenetic experiments or achieving clinical diagnosis. As claimed by the original papers describing these tools, the applied algorithms exhibit higher sensitivity (∼80%) to identify deleterious mutations. However, their specificity is low, thus a large portion of neutral predictions is false.

Nevertheless, because of the high sensitivity, those predictions, which indicate a variation deleterious, could provide valuable information for amino acid changes with unknown effects. Importantly, many of the above *in silico* tools employ information on amino acid conservation, but most parts of the ABC membrane transporters, except the nucleotide binding domains exhibit very low sequence similarity (<20% in many cases). Therefore, we investigated the applicability of two predictors, SNAP2 (26) and PROVEAN (27) employing different algorithms, to confirm if the high sensitivity of the methods is preserved and could be exploited in the case of these membrane proteins. We collected the deleterious and neutral variations in curated datasets of ABCB11, ABCC6 and CFTR, and performed the predictions on these amino acid changes (Table 1). Both PROVEAN and SNAP2 exhibited low sensitivity (60–70% of positive hits were true) for deleterious mutations in ABCB11 and ABCC6. Predictions of neutral changes were mostly at the expected low level of confidence, but SNAP2 predictions varied greatly and PROVEAN showed stable values between 40 and 50% of true positive hits. These tools also do not exhibit consequent higher sensitivity in the case of conserved regions such as NBDs (see online supplementary material for Table S2). Although we inserted both SNAP2 and PROVEAN predictions into our database, these observations clearly indicate the need of further development of these tools for ABC (membrane) proteins and we emphasize the careful utilization of *in silico* tools in designing experiments and discourage their usage for clinical applications as also pointed out by the above mentioned studies. These SNAP2 and PROVEAN predictions may be beneficial for those amino acid positions, for which there is a mutation with a known effect and the corresponding prediction shows the same effect. It is also important to note that combination of predictors does not perform better than the best predictor alone and the performance of each predictor is significantly gene dependent (37).

**Table 1:** Performance of *in silico* tools SNAP2 and PROVEAN in predicting the effect of mutations in ABC proteins as compared to curated mutations from databases

Gene	Effect	Curated	SNAP2		PROVEAN	
**ABCB11**/BSEP	Deleterious	153	98	*(64%)*^a^	100	*(65%)*
	Neutral	52	29	*(56%)*	22	*(42%)*
	Unspecified	15				
**ABCC6**/MRP6	Deleterious	182	123	*(68%)*	117	*(64%)*
	Neutral	2	1	*(50%)*	1	*(50%)*
	Unspecified	0				
ABCC7/**CFTR**	Deleterious	349	182	*(52%)*	161	*(46%)*
	Neutral	31	6	*(19%)*	14	*(45%)*
	Unspecified	629				

a The number of correctly predicted mutation effects (true positive hits).

### Deducing the effect of mutations based on sequence alignments

One of the key concepts in the development of our web application has been to facilitate the classical way of sequence-based comparative analysis of homologous proteins. Users can assess the conservation level of a given position in two or more proteins in a sequence alignment and also the conservative nature of the amino acid change. These assessments could help deducing the effect of a mutation in a given protein based on the known effect of a homologous mutation in other proteins. This is a kind of ‘low throughput’ version of *in silico* predictors, but also includes knowledge from sentences extracted from publications. However, when we aimed to compare the efficiency of our low throughput method to those of *in silico* algorithms, we noticed that a surprisingly high number of conservative changes, according to BLOSUM62 matrix, result in deleterious mutations (see online supplementary material for Table S3). In contrast, several non-conservative mutations are neutral. Still, these results are not unexpected, since an amino acid contributes to the protein function in a structural environment. A simple example, when an amino acid is located on the protein surface, its mutation to another amino acid with different physicochemical properties (e.g. size, charge) may not alter the folding or the function of a protein, while it would be most likely deleterious, if the change happens inside the protein or in a region participating in protein-protein interactions. It would be also plausible to include ΔΔG (the free energy difference between a wild-type and mutant protein) predictions based on structures, but there is only limited information on the structure of human ABC proteins, which is a requirement for this type of stability prediction. In addition, the performance of these methods is also limited (38, 39). Moreover, in many cases a deleterious mutation does not necessarily affect protein stability but influences the folding and routes the mutant protein to an off-pathway intermediate state, such as in the case of CFTR (40). In spite of these problems, we and users of our database are quite enthusiastic about the help provided by the application of the homology models provided by our ABCM2 framework, and their integration with knowledge from publications and sequence alignments, in deducing the effect of an amino acid change.

We evaluated the performance of a simple homology-based inference of mutations’ effects. ABCB11 and ABCC6 variations with known effects that are in locations homologous to CFTR mutations with known effects were collected (Table 2). 93% of these ABCC6 variations exhibited the same effect when compared to variations in CFTR. Even in the distant family member, ABCB11, the effects of 86% of mutations coincided with those in CFTR. This suggests that a simple predictor utilizing only sequence alignments to map locations to a protein, whose variants have been well annotated, would already exhibit high sensitivity. Interestingly, mutations in regions with lower level of conservation (e.g. transmembrane helices) could also be predicted (see next section). It is important to note that, because of the issues discussed in the previous paragraph, we did not consider whether an amino acid replacement was conservative or not. A position in the sequence was regarded generally susceptible to mutations when there was at least one deleterious variant reported for that given position. Nevertheless, this type of predictions exhibits significantly better performance than previous *in silico* methods above, which do not use existing knowledge on the effects of mutations in homologous proteins.

**Table 2:** Simple homology-based inference can be used to predict the effect of variations even in distant family members

	CFTR	
	ABCC6	ABCB11
Mutations at homologous positions	85	75
Mutations with known effect	45 (100%)	35 (100%)
Matching effects	43 (96%)	30 (86%)

### Distribution of variations over different domains

Protein–protein interaction interfaces have been shown to be prone to mutations, and this can also be expected in the case of intramolecular domain-domain interactions. It has been demonstrated that disease causing mutations in ABCC6 are clustered at the domain-domain interfaces between NBD1 (nucleotide binding domain 1) and NBD2, and between NBDs and the coupling helices (41). Moreover, this phenomenon is also observable in the case of other membrane proteins. Many disease-causing mutations in members of the human CLC family of chloride channels reside at the interface of the cytoplasmic cystathionine beta-synthase and the transmembrane domains (42). Therefore we investigated the distribution of mutations for ABCB11, ABCC6 and CFTR (Table 3), in this paper with a larger number of collected deleterious mutations as compared to earlier studies.

**Table 3:** Distribution of deleterious mutations in different regions of ABC proteins

Gene	Region	Ratio of mutated positions^a^	
ABCB11	TMD1_EL	10%	(8/83)
ABCB11	TMD2_EL	8%	(3/40)
ABCB11	TMD1_TH	9%	(12/133)
ABCB11	TMD2_TH	7%	(10/137)
ABCB11	TMD1_CL	11%	(12/106)
ABCB11	TMD2_CL	7%	(7/107)
ABCB11	NBD1	16%	(39/237)
ABCB11	NBD2	11%	(26/239)
ABCB11	CL	8%	(20/239)
ABCC6	TMD1_EL	3%	(1/29)
ABCC6	TMD2_EL	4%	(1/23)
ABCC6	TMD1_TH	10%	(12/121)
ABCC6	TMD2_TH	5%	(6/117)
ABCC6	TMD1_CL	15%	(18/121)
ABCC6	TMD2_CL	13%	(16/126)
ABCC6	NBD1	15%	(34/225)
ABCC6	NBD2	16%	(38/235)
ABCC6	CL	9%	(29/320)
CFTR	TMD1_EL	39%	(11/28)
CFTR	TMD2_EL	14%	(5/35)
CFTR	TMD1_TH	30%	(36/121)
CFTR	TMD2_TH	15%	(18/117)
CFTR	TMD1_CL	15%	(18/121)
CFTR	TMD2_CL	25%	(32/126)
CFTR	NBD1	25%	(55/224)
CFTR	NBD2	18%	(43/234)
CFTR	CL	14%	(66/474)

a Number of positions with mutations/length of region.

TMD: transmembrane domain, EL: extracellular loop, CL: cytoplasmic loop, NBD: nucleotide binding domain, TH: transmembrane helix.

The disease causing mutations were found to be distributed relatively evenly over the sequence of the ABCB11 protein. There is one exception, namely a slight enrichment can be observed for mutations in NBD1. In ABCC6, the extracellular loops and also the helices in the second transmembrane domain (TMD) are also depleted in deleterious mutations. However, the N-terminal transmembrane helices, the C-terminal intracellular loops and the nucleotide binding domains are overrepresented by variations with serious effects. Intriguingly, in CFTR, for which the most disease causing mutations among ABC proteins are known, deleterious mutations are overrepresented in the extracellular loops and the transmembrane helices of TMD1, and in the intracellular loops of TMD2. Moreover, NBD1 is enriched in CF-causing mutations as compared to NBD2. Although based on these data and our current knowledge on structure/function relationship of ABC proteins, it is difficult to draw solid conclusions, sensible notes can be made based on the hypothesis that a region susceptible to mutations is of high importance, e.g. for function or folding. The observation, that there is no enrichment of mutations in ABCB11 transmembrane regions may be attributed to the absence of a classical, well-defined substrate binding pocket because of the wider spectrum of hydrophobic substrates. In contrast, in the two ABCC subfamily members disease causing mutations are enriched in the N-terminal TMD1, which may be attributed to either the asymmetric function of NBDs or to a sensitive folding process. It is characteristic for ABCC proteins that ATP is bound to NBD1 but the hydrolysis is diminished, in contrast to the hydrolysis of ATP at the NBD2 site. This asymmetry most likely also causes asymmetry in the transmembrane domains. In addition, these ABCC proteins transport or conduct charged molecules, thus they possess charged residues in their transmembrane helices. For example, many arginine residues are present in the sixth transmembrane helix (TH6) in CFTR TMD1 known to take part in forming the chloride channel. The presence of charged residues, which are located in the hydrophobic region of the membrane bilayer, may exert a delicate balance during the folding process of TMD1 which accordingly becomes more prone to mutations. In addition, the N-terminal half may also be important serving as a scaffold for the folding of the C-terminal half of these proteins. Interestingly, the removal of the full NBD2 in the C-terminal part maintains CFTR folding and maturation, while many point mutations in the N-terminal half abrogate CFTR (43, 44).

### Sparse information on non-coding regions

 Since variations in non-coding regions may also exhibit deleterious effects on functional protein expression, we aimed to include mutations located in non-coding regions of ABC genes into our database. We identified 1 SNP for *ABCB11* (BSEP) in ClinVar, 2 SNPs in ClinVar and 25 in LOVD for *ABCC6* (MRP6) and 154 SNPs for *CFTR* in non-coding regions (Clinvar, CFTR and CFTR2). Most of these are alterations in introns and a fraction of them are splice site mutations and data are sparse on single nucleotide variants located in well-defined regulatory regions or with transcriptional regulatory effects. For example, only one variation has been reported for the *ABCC6* promoter region decreasing PLAG transcription factor binding (45, 46). Six and one alterations in the promoter region were found for *CFTR* from ClinVar and CFTR2, respectively.

In order to facilitate the understanding of variations in *cis*-regulatory regions, we also started to collect information on transcription regulatory sites. In order to assess the associated resources, as a first step we collected transcription factors and their binding locations influencing the transcription of *ABCB11*, *ABCC6* and *CFTR*. Data mining of the papers with experiments using human systems and reporting direct regulation resulted in only a few exact locations for transcription factor binding sites. These include FXR, LRH-1 and Nrf2 binding sites in the *ABCB11* promoter (47–50) and NF-κB, Sp1/Sp3 and PLAG1/PLAGL1 responsive elements in the *ABCC6* promoter (51, 52). There are several characterized cis-regulatory regions of *CFTR*, such as in introns 1 and 11, recruiting transcription factors including forkhead box A1/A2 (FOXA1/A2), hepatocyte nuclear factor 1 (HNF1) and caudal-type homeobox 2 (CDX2) (53, 54). In addition, distant *CFTR* regulatory regions are also described in detail (55). However, we decided not to include this information into the current version of our database because of their low resolution. Other publicly available resources do not contain related data for insertion into ABCM2. For example, the DECODE (DECipherment Of DNA Elements) database, which combines ABiosciences' proprietary database, QIAGEN’s Text Mining Application, and also data from the UCSC Genome Browser, does not list any of the transcription factors we collected from publications (http://www.sabiosciences.com/chipqpcrsearch.php). Moreover, it lists transcription factors for these ABC proteins that we could not confirm with publications.

### Updated database and web interface

 Since we have inserted data reflecting the genomic level into our protein-centered database, new tables and relations had to be introduced. New tables were also inserted for curated mutations (e.g. from ClinVar, ABCC6 LOVD, CFTR2 and reviews) and also for the effects of all possible mutations predicted by SNAP2 and PROVEAN. At this moment this level of data isolation, namely storing different types of mutations in different tables, seems to be sufficient. However, in the future with more data with different types, the database may be split into several specialized databases (e.g. into separate databases for genomic and protein level data).

The new data types required slight changes in the web application interface. One set includes the presentation of novel data (e.g. the predicted effects of mutations, mutations in non-coding regions; see online supplementary material for Figure S1), which is listed on the web page in detail. We also aimed to supplement the mutations and sentences identified by text mining earlier with information on their effects on phenotype, from other databases and *in silico* predictions (see online supplementary material for Figure S2). An important set of changes includes novel search possibilities, such as querying variations at the DNA level and submitting a list of genomic and amino acid positions for batch queries (see online supplementary material for Figure S3).

Further data were also deposited in our database. First, a structure of the ABCG5-ABCG8 heterodimer has been published recently (56). We included this structure and our ABCG2 homology model (57) into our web application. This new transmembrane fold of the ABCG subfamily members now can be employed to visualize spatial locations of amino acids in ABCG proteins and interpret their effects in a 3 D context. Second, a refined classification of CF mutations was linked to variations of CFTR. Traditional categories have been numbered from I to VI, including mutations resulting in premature termination codons (PTC), misfolding, impaired regulation, altered channel conductance, decreased abundance and destabilization in post-ER compartments, respectively. Since many mutations exhibit several of these listed effects and could not be included clearly into a category, thus combinatorial categories have been created by Veit *et al*. (58). We included these information rich annotations from their publication.

## Conclusion

Although many ABC proteins are responsible for diseases and widely studied, there is a significant lack of data on the effects of sequence variations even in large disease focused databases. Importantly, the effects of a significant portion of the mutations are not annotated. This can be attributed most likely both to the attitude of researchers (59) and to the lack of knowledge on the connection of mutations to disease phenotypes and. For example, a large number of CFTR mutations is identified in patients in the presence of other mutations with unknown effects on the same or on the other chromosome, making impossible to dissect the contribution of these variations to the phenotype. Whilst the curated information in LSDBs is related mostly to the phenotypic effects and the predictions can indicate only deleteriousness, it would be important to decipher the effects at the cellular level (such as folding, trafficking and function) of the variations. At present, it does not seem feasible to predict these effects *in silico* with high confidence.

There is a trend to store data on variations in large and centralized databases (e.g. ClinVar), which has several advantages. For example, if a central site contains all known variations for a gene, querying only this database is sufficient instead of visiting several LSDB sites. In addition, depositors do not need to create and maintain an own LSDB, the data on variations and tools for querying and analysis. However, we faced the retirement of LOVD databases hosted by the NIH and data were not available in ClinVar a month after stopping the service, at the time of this manuscript submission. This warns about the possibility that the maintainer of a central database can decide any time to close a site temporarily or even permanently. This phenomenon could effectively be prevented by a federated approach such as Cafe Variome (60), containing a central server for efficient querying and several independently accessible locus-specific data stores. This type of approaches can fulfill the roles expected from a central site, and also maintain the distribution of data with higher persistency and help in avoiding even temporary inaccessibility. Importantly, for this setup, standards, such as in the case of Cafe Variome, should be defined accurately to allow the communication between the nodes for query, access control and data exchange management (60). In addition, the web application on each node could be tuned according to the field of the given gene, similarly to how our web application includes sequence alignments and structures of ABC proteins. Since implementation of such extra features specific for a subset of genes is not rational at a central database, we will surly witness the development and emergence of LDBDs in the next decades (59).

Our current results indicate that combining available information on the effects of mutations with sequence alignments is still an important and useful approach in predicting the outcome of variations with unknown effects in homologous proteins (Table 2). Our web application can significantly aid this prediction process. In addition, we initiated the inclusion of DNA level information on ABC proteins and implemented batch query possibility that also promote further developments for personalized medicine.

## Supplementary data

Supplementary data are available at *Database* Online.

## Supplementary Material

Supplementary Data
