# Supplementary material for: ABCMdb reloaded: updates on mutations in ATP binding cassette proteins
Source: Database (Oxford). 2017 Mar 18;2017:bax023. doi: 10.1093/database/bax023 (PMC5467578; doi:10.1093/database/bax023)

ABCMdb reloaded: updates on mutations in ATP Binding Cassette proteins

Hedvig Tordai, Kristóf Jakab, Gergely Gyimesi, Kinga András, Anna Brózik,

Balázs Sarkadi, Tamás Hegedűs

Supporting Information

Table S1. Variations queried from ClinVar. Genetic alterations were filtered and categorized as missense, nonsense, and synonymous mutations. Genes were ordered by the number of variations. D: deleterious, N: neutral, ?: unspecified.

gene	all	coding	unique	synonymous	missense				nonsense			
				total	D	N	?	total	D	N	?	total
**CFTR**	901	747	**731**	**38**	100	10	440	**550**	52	0	91	**143**
ABCA4	476	372	**370**	**52**	28	10	234	**272**	7	0	39	**46**
ABCC9	115	91	**91**	**33**	14	2	38	**54**	1	1	2	**4**
ABCC8	66	49	**49**	**16**	24	3	2	**29**	4	0	0	**4**
ABCD1	50	43	**43**	**4**	28	0	5	**33**	6	0	0	**6**
ABCA3	27	26	**26**	**7**	6	1	11	**18**	1	0	0	**1**
ABCC2	25	20	**20**	**5**	4	6	3	**13**	2	0	0	**2**
**ABCC6**	23	21	**21**	**1**	13	3	1	**17**	3	0	0	**3**
ABCB4	21	19	**19**	**2**	6	2	7	**15**	2	0	0	**2**
ABCC1	21	5	**5**	**2**	0	0	3	**3**	0	0	0	**0**
ABCA1	15	14	**14**	**0**	12	0	1	**13**	1	0	0	**1**
**ABCB11**	15	14	**14**	**4**	2	3	3	**8**	2	0	0	**2**
ABCG8	13	12	**12**	**1**	3	1	3	**7**	4	0	0	**4**
ABCB7	11	9	**9**	**2**	5	2	0	**7**	0	0	0	**0**
ABCG5	10	9	**9**	**1**	2	0	2	**4**	4	0	0	**4**
ABCB6	9	9	**9**	**0**	6	0	2	**8**	1	0	0	**1**
ABCA12	8	8	**8**	**0**	7	0	0	**7**	1	0	0	**1**
ABCB1	5	5	**5**	**1**	1	3	0	**4**	0	0	0	**0**
ABCG2	5	5	**5**	**0**	0	0	2	**2**	1	0	2	**3**
TAP1	3	3	**3**	**0**	1	2	0	**3**	0	0	0	**0**
TAP2	3	3	**3**	**0**	0	2	0	**2**	0	1	0	**1**
ABCC4	1	1	**1**	**0**	0	0	1	**1**	0	0	0	**0**
ABCC5	1	1	**1**	**0**	0	0	1	**1**	0	0	0	**0**
ABCD3	1	1	**1**	**0**	0	0	1	**1**	0	0	0	**0**
ABCD4	1	1	**1**	**0**	1	0	0	**1**	0	0	0	**0**
ABCG4	1	1	**1**	**0**	0	0	1	**1**	0	0	0	**0**
ABCA5	1	0	**0**	**0**	0	0	0	**0**	0	0	0	**0**
ABCA10	0	0	**0**	**0**	0	0	0	**0**	0	0	0	**0**
ABCA13	0	0	**0**	**0**	0	0	0	**0**	0	0	0	**0**
ABCA2	0	0	**0**	**0**	0	0	0	**0**	0	0	0	**0**
ABCA6	0	0	**0**	**0**	0	0	0	**0**	0	0	0	**0**
ABCA7	0	0	**0**	**0**	0	0	0	**0**	0	0	0	**0**
ABCA8	0	0	**0**	**0**	0	0	0	**0**	0	0	0	**0**
ABCA9	0	0	**0**	**0**	0	0	0	**0**	0	0	0	**0**
ABCB10	0	0	**0**	**0**	0	0	0	**0**	0	0	0	**0**
ABCB5	0	0	**0**	**0**	0	0	0	**0**	0	0	0	**0**
ABCB8	0	0	**0**	**0**	0	0	0	**0**	0	0	0	**0**
ABCB9	0	0	**0**	**0**	0	0	0	**0**	0	0	0	**0**
ABCC10	0	0	**0**	**0**	0	0	0	**0**	0	0	0	**0**
ABCC11	0	0	**0**	**0**	0	0	0	**0**	0	0	0	**0**
ABCC12	0	0	**0**	**0**	0	0	0	**0**	0	0	0	**0**
ABCC3	0	0	**0**	**0**	0	0	0	**0**	0	0	0	**0**
ABCD2	0	0	**0**	**0**	0	0	0	**0**	0	0	0	**0**
ABCG1	0	0	**0**	**0**	0	0	0	**0**	0	0	0	**0**

Table S2. Performance of SNAP2 and PROVEAN does not increase largely and consequently for the conserved nucleotide binding domains as compared to curated mutations from databases.

NBD:

gene	effect	curated	SNAP2		PROVEAN	
**ABCB11**/BSEP	deleterious	*75*	*52*	*(69%)**	*54*	*(72%)*
	neutral	*18*	*7*	*(39%)*	*6*	*(33%)*
	unspecified	*5*				
**ABCC6**/MRP6	deleterious	*79*	*55*	*(70%)*	*56*	*(71%)*
	neutral	*0*				
	unspecified	*0*				
ABCC7/**CFTR**	deleterious	*121*	*63*	*(52%)*	*70*	*(58%)*
	neutral	*14*	*4*	*(29%)*	*6*	*(43%)*
	unspecified	*213*				

TMD:

gene	effect	curated	SNAP2		PROVEAN	
**ABCB11**/BSEP	deleterious	57	37	*(65%)*	36	*(63%)*
	neutral	26	17	*(65%)*	10	*(38%)*
	unspecified	8				
**ABCC6**/MRP6	deleterious	71	50	*(70%)*	49	*(69%)*
	neutral	1	0	*(0%)*	0	*(0%)*
	unspecified	0				
ABCC7/**CFTR**	deleterious	151	91	*(60%)*	68	*(45%)*
	neutral	7	1	*(14%)*	4	*(57%)*
	unspecified	279				

** The number of correctly predicted mutation effects (true positive hits)*

Table S3. Significant portion of conservative and non-conservative amino acid replacements can lead to deleterious and neutral changes, respectively. Variants with premature stop and silent amino acid changes were not counted.

**a.a. change:**	**conservative**				**non-conservative**			
**effect:**	**deleterious**		**neutral**		**deleterious**		**neutral**	
ABCB11/BSEP	51%	(25/49)	49%	(24/49)	80%	(105/131)	20%	(26/131)
ABCC6/MRP6	100%	(39/39)	0%	(0/39)	98%	(117/120)	3%	(3/120)
ABCC7/CFTR	87%	(48/55)	13%	(7/55)	94%	(206/218)	6%	(12/218)

Figure S1. The major changes in the web interface associated with novel data, such as predicted effect of mutations by SNAP2 and PROVEAN, effect of mutations collected from databases (top panel), and mutations in non-coding regions (bottom panel).

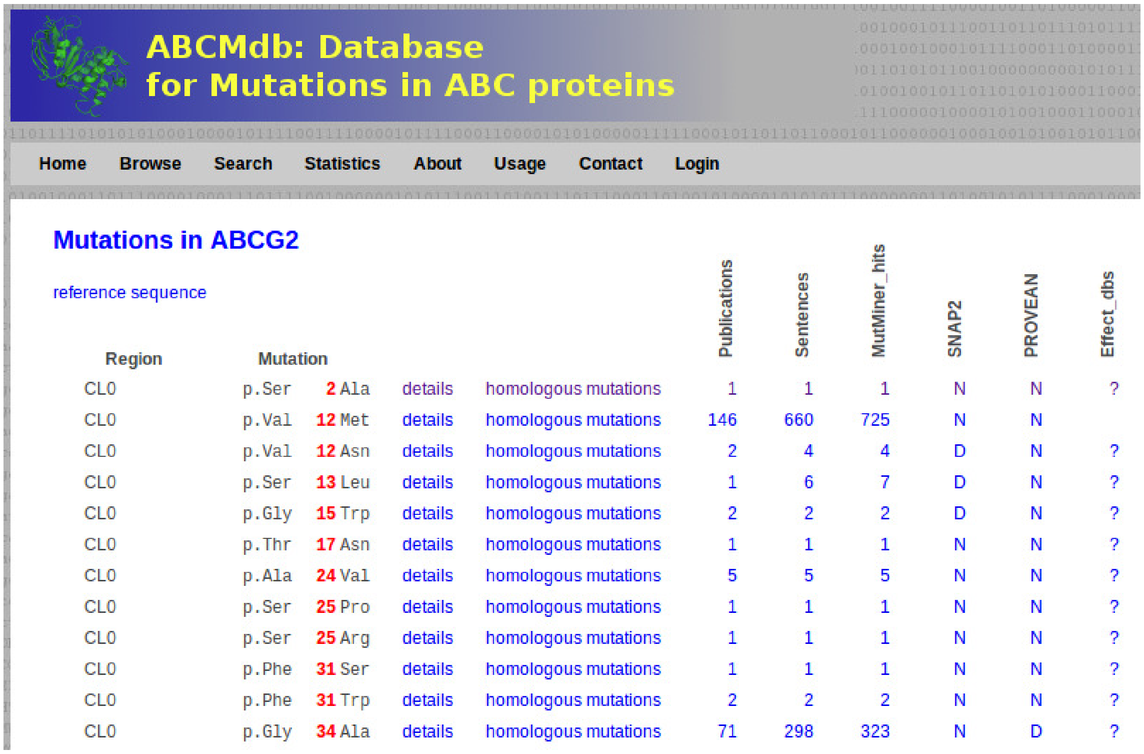

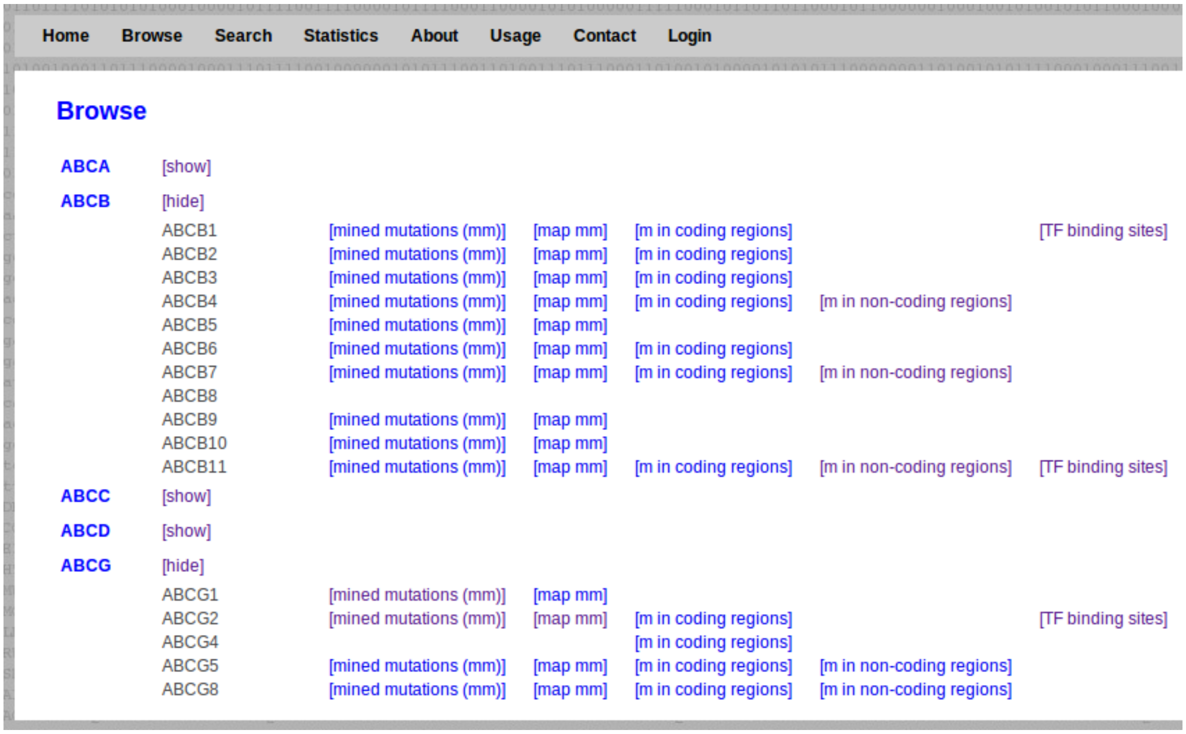

Figure S2. Mutations identified in an automatic way in full text papers are connected to entries in other data sources such as ClinVar and LSDBs and to *in silico* predictions by SNAP2 and PROVEAN to deliver as much information on phenotype as possible.

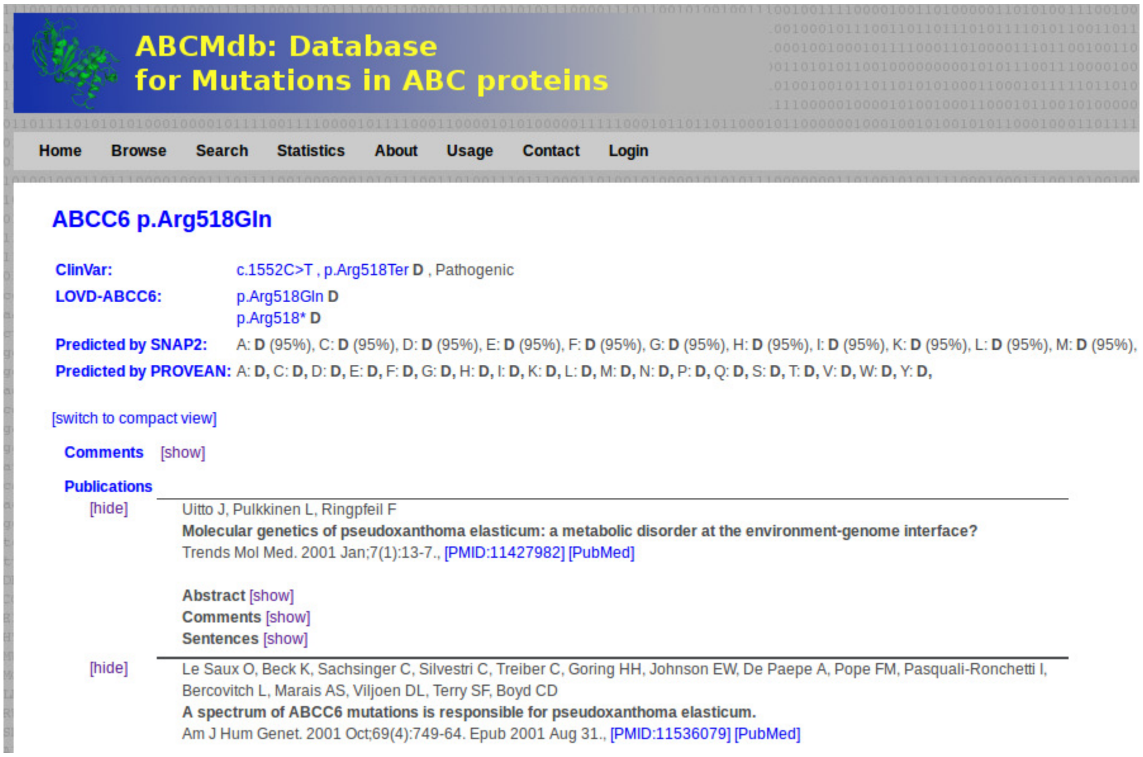

Figure S3. Querying variations at the DNA level and batch queries are also novel additional changes in the web interface.

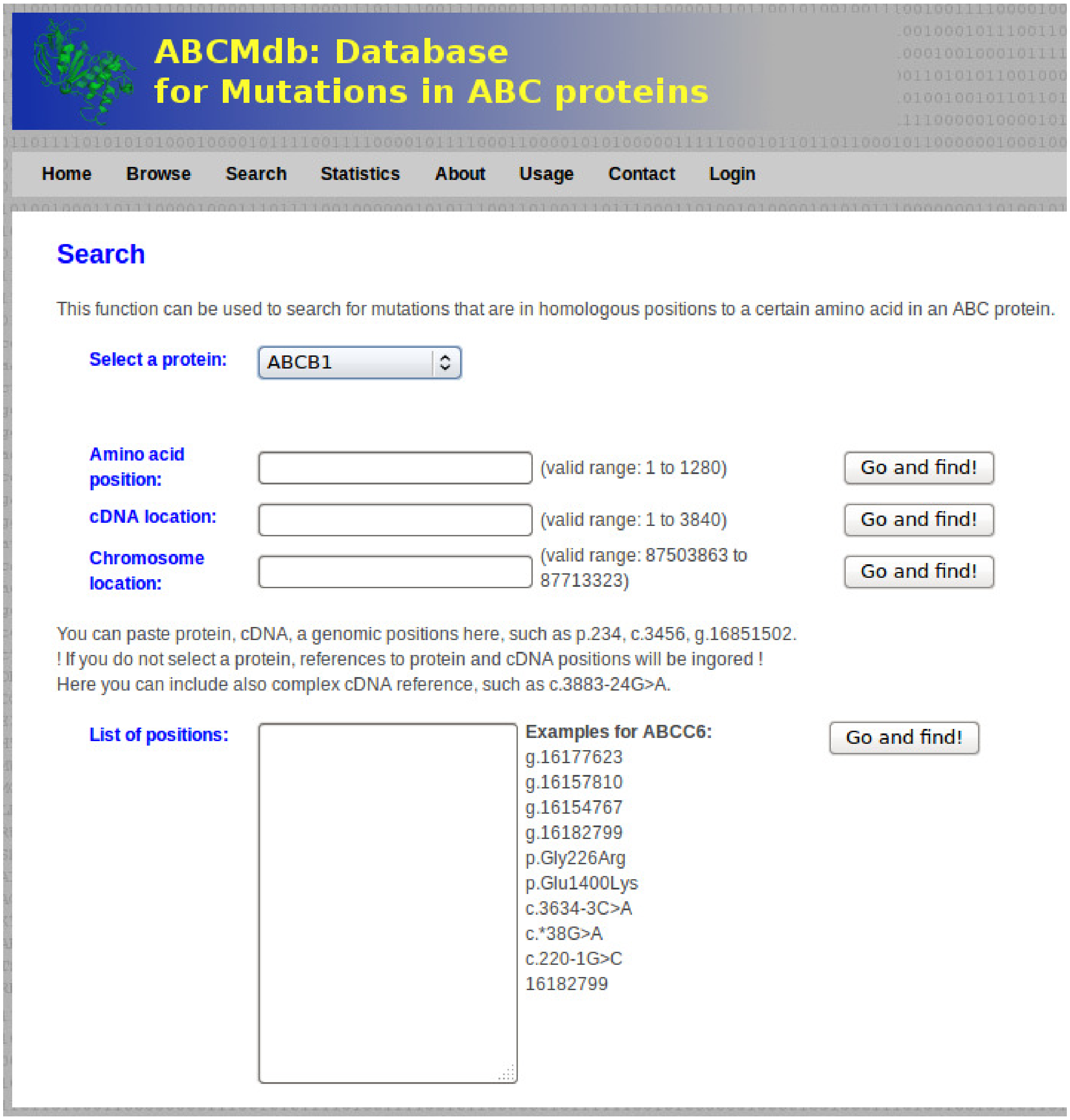

Supplement: Supplementary Data [file bax023_Supp.pdf]
